# Use of a combined sublay–onlay “sandwich” technique for abdominal incisional hernia repair: a case report

**DOI:** 10.3389/fsurg.2026.1814872

**Published:** 2026-07-07

**Authors:** Xing-Kai Kang, Bin Wu, Jing Wang, Yi-Zhou Shao, Tao Wang

**Affiliations:** 1 Department of Hepatobiliary Surgery, The Second Affiliated Hospital of Jiaxing University, Jiaxing, Zhejiang Province, China; 2 Zhejiang Chinese Medical University, Hangzhou, Zhejiang Province, China

**Keywords:** abdominal wall reconstruction, case report, giant incisional hernia, incisional hernia, mesh repair, polypropylene mesh, sandwich technique

## Abstract

An abdominal incisional hernia is one of the most common complications of abdominal surgery. Giant incisional hernias often make it difficult to choose an optimal treatment plan because of the large abdominal defects. This article reports the case of a large incisional hernia with an abdominal wall defect width of approximately 12 cm. We implemented a double-layer polypropylene patch repair technique combining sublay and onlay placement—a “sandwich” technique. The patient recovered smoothly postoperatively, and the 6-month follow-up showed good results for abdominal wall repair without recurrence. The “sandwich” technique was demonstrated to be a reliable strategy, and this case provides feasible ideas for doctors to treat such patients. This approach provides a solution for giant incisional hernias by overcoming the mechanical limitations associated with traditional single-layer mesh repairs, thereby ensuring better abdominal wall stability.

## Introduction

Abdominal incisional hernia is one of the most common complications of abdominal surgery, caused by poor healing or rupture of the fascial layer deep in the original surgical incision. Although the skin and subcutaneous tissue may have healed in a single stage, a defect in the fascial layer under the continuous pressure of the abdominal cavity can cause the abdominal contents to bulge outward. An incisional hernia not only causes a local abdominal wall mass and discomfort, affecting the patient's quality of life, but may also lead to serious complications, such as intestinal obstruction, entrapment, and even strangulation, posing a threat to the patient's health (1).

The European Hernia Society (EHS) abdominal incisional hernia classification system is widely used internationally, which focuses on the transverse width (W) of abdominal wall defects: W1 (<4 cm), W2 (4–10 cm), and W3 (>10 cm) (2). The patient described in this report had an abdominal wall defect measuring approximately 12 cm; therefore, it was classified as W3 in the EHS classification.

For larger incisional hernias, traditional direct suture repair can have a recurrence rate of up to 30%–50% due to high tension (3). The application of patch materials greatly improves prognosis and has become a common method for repairing medium- and large-sized incisional hernias (4). Commonly utilized techniques for mesh placement include sublay, onlay, and intraperitoneal onlay mesh (IPOM). Currently, there is no consensus on the optimal surgical approach for incisional hernia. The sublay technique is considered to offer favorable biomechanical advantages and good tissue compatibility. The onlay technique is more superficial and technically straightforward but may carry a higher risk of seroma formation. The intraperitoneal onlay mesh (IPOM) technique requires the use of anti-adhesive mesh and is associated with higher costs. Furthermore, research on the use of double-layer mesh remains limited (5, 6).Individualized treatment decisions must be based on the patient's specific situation, including defect size, location, abdominal adhesion status, and overall condition.

This case report presents the diagnosis and treatment process of a patient with a giant incisional hernia and an abdominal wall defect of 12 cm in width. The selected surgical approach was a sublay combined with onlay double-layer polypropylene patch repair—a “sandwich” technique. After active treatment, the patient achieved a good prognosis, with no recurrence to date.

## Case report

**Figure 1:**
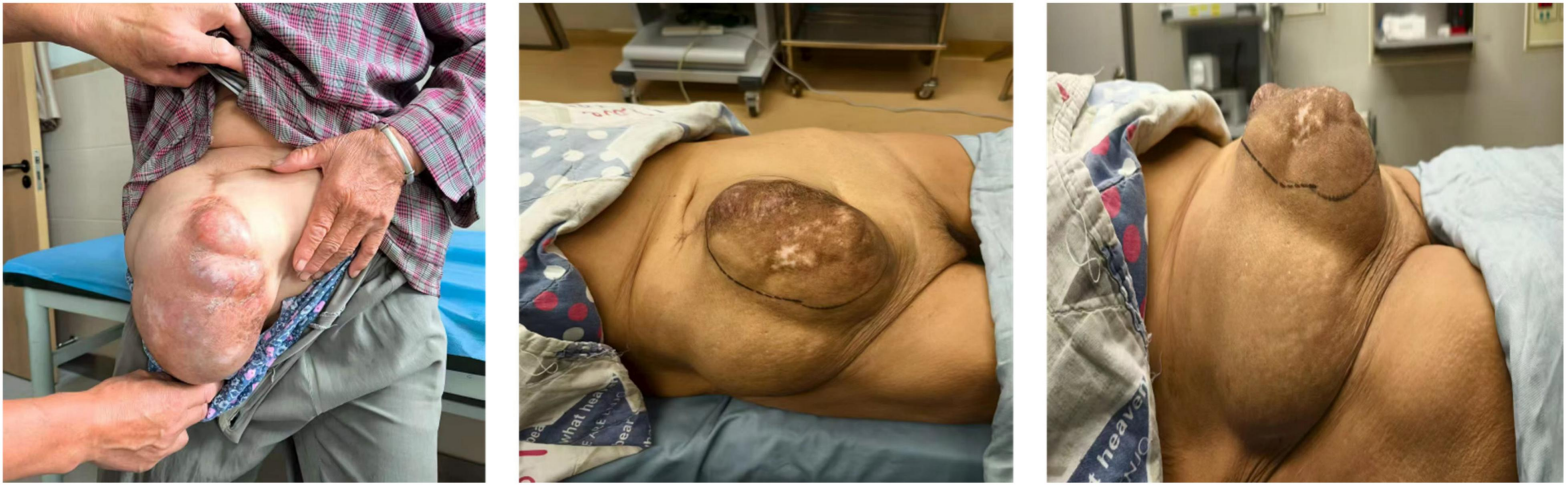
Preoperative images of patients in standing, lateral, and supine positions.

**Figure 2:**
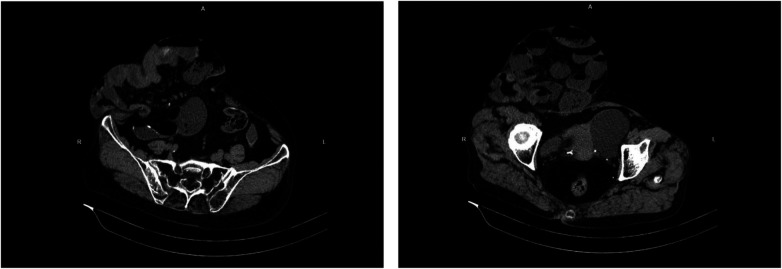
Preoperative CT image of the patient.

The patient, a 76-year-old female, presented with a massive abdominal lump that had increased in size over 5 years, accompanied by pain for 1 week. The patient was admitted to the hospital for treatment. The patient's height was 160 cm, weight was 63 kg, and body mass index (BMI) was 24.6 kg/m^2^. The patient had no history of smoking. Because of the lack of pain caused by the lump, the patient did not seek treatment until now. She had previously undergone open local intestinal resection due to strangulated intestinal obstruction. There was no history of surgical site infection (SSI) following that procedure. A physical examination revealed a basketball-sized lump in the lower right abdomen, measuring approximately 35 × 18 cm when standing and not fully retractable when lying down. Tenderness was observed in the lower right abdomen, with no rebound pain or abdominal muscle tension. In addition, no varicose veins in the abdominal wall were observed, and there was no abnormal vascular pulsation (Figure 1). Computed tomography (CT) revealed an abdominal hernia with intestinal obstruction and intestinal tubes protruding through the right lower abdominal hernia (Figure 2).

**Figure 3:**
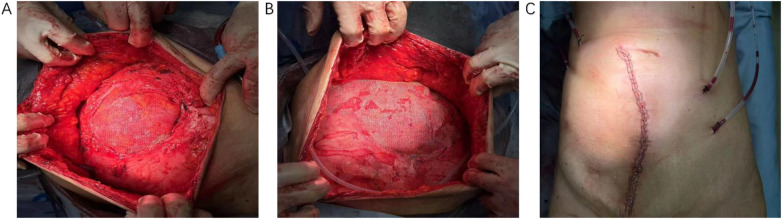
**A** shows the patch placed below the muscle layer. **B** shows the patch placed above the muscle layer. **C** shows the surgery after completion.

We performed abdominal incisional hernia repair 8 days after admission. The patient presented with a 10-year history of hypertension but was otherwise healthy, staged as ASA II. Based on pre-operative CT findings suggesting mechanical obstruction and the anticipated need for complex adhesiolysis, a direct open approach (laparotomy) was chosen. During surgery, we first removed the scar tissue from the original incision, which was beneficial for the healing and rehabilitation of the new incision. A flexible incision was made along the original midline of the abdomen with a length of approximately 20 cm. During the operation, the hernial sac was dissected 1 cm below the navel and 3 cm above the pubic bone. The defect length was approximately 12 cm, and the width was approximately 7 cm. Opening of the hernia sac revealed a large amount of omentum and the small intestinal tract, with adhesions between the intestinal tract and the abdominal wall. Surgical exploration revealed that the obstruction was multifactorial, involving both the entrapment of intestinal tract within the hernia defect and adhesions. After loosening the adhesions of the intestinal tract, it was completely retracted into the abdominal cavity. Two negative-pressure drainage tubes were placed intraperitoneally to facilitate the drainage of intra-abdominal fluid. The peritoneum was sutured using a 3–0 antibacterial absorbable suture. A hernia patch approximately 25 × 22 cm in size was placed under the anterior sheath of the rectus abdominis muscle. Due to the significant abdominal wall defect and excessive tension of the surrounding tissues, primary closure of the anterior rectus sheath was not feasible. Consequently, the defect was managed using a bridging repair technique with mesh. Next, another hernia patch approximately 22 × 20 cm in size was placed between the anterior sheath of the rectus abdominis muscle and the subcutaneous tissue. To prevent fluid accumulation, two negative-pressure drainage tubes were placed above the patch and subcutaneous tissue. The incision was then closed (Figure 3). The total operative time was 4 h and 13 min. Prophylactic antibiotics were administered intraoperatively according to standard protocols. Postoperatively, no signs of surgical site infection (SSI), such as erythema or purulence, were noted. Although a localized subcutaneous seroma was observed at the incision site in the early postoperative period, it was managed conservatively and gradually resolved during follow-up.

**Figure 4:**
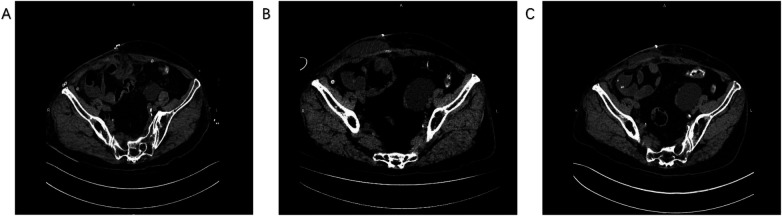
**A** is the abdominal CT scan on the third day after surgery, **B** is the abdominal CT scan on the seventh day after surgery, and **C** is the abdominal CT scan on the fourteenth day after surgery.

On days 3, 7, and 14 after surgery, the abdominal CT scans were re-examined (Figure 4). The drainage tube volumes and postoperative inflammatory indicators are shown in Table 1. Drain 1: left intraperitoneal drain; Drain 2: right intraperitoneal drain; Drain 3: left subcutaneous drain; Drain 4: right subcutaneous drain. The patient was discharged on the day 15 after surgery.

**Table 1:** Postoperative drainage fluid and inflammatory indicators.

Postoperative time/day	1	3	5	7	10
Drainage volume 1: Left Intraperitoneal (mL)	210	100	50	50	5
Drainage volume 2: Right Intraperitoneal (mL)	40	70	50	15	0
Drainage volume 3: Left Subcutaneous (mL)	50	80	100	2	/
Drainage volume 4: Right Subcutaneous (mL)	30	50	50	60	40
white blood cell count (10⁹/L)	15.07	13.81	4.81	5.59	5.12
PCT (ng/mL)	0.232	0.316	0.237	0.154	0.083

## Discussion

Abdominal incisional hernia is often caused by an unhealed fascia or muscle layer of the abdominal wall after abdominal surgery (7). Surgery is the main treatment option for incisional hernias, including open, laparoscopic, and assisted surgery (8). Currently, open-incision hernia repair is the primary method, and the main surgical techniques include onlay, inlay, sublay, and intraperitoneal onlay mesh (IPOM). Personalized surgical plans must be developed for the treatment of large incisional hernias. Currently, the optimal surgical approach for repairing large incisional hernias remains controversial. Limited research has been conducted on the surgical methods for placing double-layer hernia patches. The main differences lie in the lack of uniformity in patch materials and the hierarchy of patch placement (9, 10). This case report describes the diagnosis and treatment of a large incisional hernia in the abdominal wall. The width of the patient's defect was 12 cm, classified as W3 according to the EHS classification. We developed a personalized treatment plan for the patient: sublay combined with onlay “sandwich” technology.

Preoperative evaluation is the cornerstone of developing a treatment strategy. Previous abdominal surgery (intestinal resection) inevitably damages the integrity of the abdominal fascial layer. Patients may experience poor healing, infection, or simple mechanical traction after surgery, resulting in insufficient healing. Under continuous pressure in the abdominal cavity, the weak points gradually rupture, forming a hernial ring. After abdominal surgery, the incomplete posterior heath of the rectus abdominis muscle and its relatively high intra-abdominal pressure are one of the most common sites of incisional hernia (11). The treatment of incisional hernia is comprehensive, and actively addressing the primary cause is fundamental for preventing long-term recurrence. Therefore, we need to systematically evaluate whether underlying conditions, such as chronic coughing, long-term constipation, and obesity, lead to sustained or intermittent increases in intra-abdominal pressure. In men, it is necessary to assess whether there is an increased risk with smoking and prostate hyperplasia (12). Preoperative CT examination helps clarify the size and texture of the hernia ring and can be used to evaluate abdominal wall muscle function. In addition, CT can non-invasively and accurately measure the transverse and sagittal diameters of the hernia ring, enabling three-dimensional reconstruction (13). In the current patient, while CT evaluation confirmed the size of the defect, it was particularly important to assess the adhesion points of the hernia contents (mostly intestinal and omental) to the abdominal wall and the hernia sac. The adhesions identified on imaging influenced our final surgical plan, as laparoscopic separation of dense adhesions significantly increased the risk of intestinal injury. Open surgery provides the best exposure of the surgical field, allowing surgeons to perform precise and safe lysis of adhesions under direct visualization.

Finally, we developed a personalized sublay–onlay “sandwich” technique for this patient's treatment. This is a double-layer patch-repair technique. Previous studies have shown that the sublay technique, placing the patch between the vascularized rectus abdominis muscle and peritoneum, conforms to biomechanical principles: the patch adheres tightly to the muscle under intra-abdominal pressure, not only fixing reliably, but also promoting the growth of connective tissue into the patch pores, forming a sturdy “steel cement” structure. Postoperative recurrence and surgical site infection rates have been reported to be relatively low (14). According to Rudmik et al., recurrence rates vary significantly across surgical techniques: the inlay technique exhibits recurrence rates as high as 48%, followed by the onlay technique at 14%, while sublay and IPOM techniques demonstrate the most favorable outcomes with rates ranging from 4.5% to 10% (15). In this case, the patient had a large abdominal wall defect. Moreover, the patient was elderly and had weak abdominal muscles. If only a single-layer sublay patch repair had been implemented, the repaired abdominal wall may not have been able to withstand tension during long-term activities. After a thorough preoperative discussion, we decided that a more proactive reinforcement strategy was necessary. Additionally, while the component separation technique (CST) could potentially facilitate primary fascial closure, it was deemed excessively traumatic for this patient, carrying a higher risk of extensive wound complications. Regarding IPOM, the high cost of anti-adhesive composite meshes presents a significant economic burden.Furthermore, we anticipated that a laparoscopic IPOM approach would be technically challenged by the patient's adhesions from previous surgery. Consequently, these factors also dictated our choice of polypropylene mesh over a composite mesh. Therefore, we chose an open sublay and onlay double-layer polypropylene patch repair procedure for this patient. Theoretically, the double-layer patch structure provides better mechanical properties; sublay and onlay patches work together to distribute the tension borne by the abdominal wall across a wider range of healthy tissues, thereby minimizing the risk of long-term patch protrusion and recurrence. In addition, the patch is placed outside the peritoneum, without direct contact with visceral organs in the abdominal cavity; therefore, there is no need to use expensive composite anti-adhesion patches, significantly reducing medical costs and avoiding potential complications associated with them (16). The patient and their family were fully informed of the surgical plan, the rationale for the sandwich technique, and the potential alternative procedures before providing informed consent.

In this case, we finely separated the adhesions between the intestinal tract, omentum, and hernia sac wall, and used absorbable sutures to close the peritoneum. Subsequently, two planes were created for patch placement: the sublay and onlay gaps. Many nerves innervate the abdominal wall muscles, and the blood vessels that nourish them pass through the sublay gap, which should be protected as much as possible during dissection. The onlay gap usually requires a minimum distance of 5 cm beyond the edge of the hernial ring to ensure that the patch can be smoothly spread and fixed (17). Owing to the large abdominal wall defect and high surrounding tissue tension, we chose to use bridging repair technique. However, the incidence of postoperative serum swelling, the risk of patch protrusion, and hernia recurrence is higher (18). Therefore, it is important to stop bleeding during surgery, ensure smooth drainage after surgery, and take measures, such as postoperative abdominal pressure bandaging, to prevent complications, such as serum swelling, to the greatest extent possible.

## Conclusion

The treatment of abdominal incisional hernias is expected to become more precise and minimally invasive, providing patients with diverse and optimized options. Through the successful treatment of this patient, we realized the value of open sublay plus onlay double-layer patch repair surgery for specific patients. The surgical advantages include high safety (direct visualization of adhesions), strong mechanical reconstruction (double-layer reinforcement), and excellent cost-effectiveness (use of ordinary polypropylene patches). This technique is particularly suitable for patients with severe adhesions, large defects, and high long-term durability requirements. However, the short-term follow-up remains a limitation, as late recurrence following giant incisional hernia repairs often manifests over several years, requiring extended clinical surveillance. We provide valuable diagnostic and treatment experiences for similar cases in clinical practice.

## Data Availability

The raw data supporting the conclusions of this article will be made available by the authors, without undue reservation.
